# Study on the coupling mechanism of nozzle structure-cavitation impact under submerged environment^☆^

**DOI:** 10.1016/j.ultsonch.2025.107632

**Published:** 2025-10-19

**Authors:** Xiuneng Li, Xide Cheng, Wenjiang Hou, Shidong Fan, Xiaofeng Guo, Xiangshu Lei, Zhenlong Fang, Yan Chen

**Affiliations:** a Sanya Science and Education Innovation Park of Wuhan University of Technology, Sanya 572025, China; b State Key Laboratory of Maritime Technology and Safety, Wuhan University of Technology, Wuhan 430063, China; c School of Naval Architecture, Ocean and Energy Power Engineering, Wuhan University of Technology, Wuhan 430063, China; d LIED, UMR 8236, CNRS, Université Paris Cité, F-75006 Paris, France; e National Key Laboratory of Nuclear Reactor Technology, Nuclear Power Institute of China Chengdu, 610041, China

**Keywords:** Water jet technology, Cavitation, Large eddy simulation, Scanning electron microscopy, Dynamic mode decomposition

## Abstract

Water jet technology has broad potential in marine engineering due to its high efficiency, energy efficiency, and environmental friendliness. This study aims to enhance the cavitation fragmentation performance of jets by integrating underwater erosion experiments with large eddy simulations to investigate the influence of petal-shaped nozzle structural parameters on cavitation impact characteristics. A multiscale analysis method integrating cavitation impact and damage mechanisms, along with a metric for cavitation intensity, has been established to quantify the cavitation impact performance of jets. Experimental and computational fluid dynamics results show that the organ-pipe nozzle (O.N_40_) with a diffusion angle of 40° has the maximum cavitation strength at target distance *L*/*D_e_* = 6. The optimum target distance *L*/*D_e_* = 8 occurs for a petal-shaped nozzle (P.N_27_) with a diffusion angle of 27°. Under these conditions, the cavitation erosion intensity is 2.29 times greater than that of O.N_40_. Microporous regions caused by bubble collapse were identified via scanning electron microscopy (SEM). Localized high-pressure phenomena emerge during cavitation cloud collapse, accompanied by outwardly propagating pressure waves. P.N_60_ cavitation bubbles exhibit a greater development rate and spatial range with higher bubble density, generating stronger cavitation impacts at the target surface. Dynamic mode decomposition (DMD) was applied to cavitation data in the shock flow field. The steady cavitation flow field was captured in the high-energy modes. The spatio-temporal evolution of microbubbles was accurately characterized by higher-order modes. This study elucidated the interaction mechanisms between nozzle structure and cavitation impact and provided theoretical foundations for optimizing nozzle designs in submerged rock-breaking applications.

## Highlights

• The jet operating parameters for the optimal cavitation shock effect in the submerged environment were obtained.
• The microscopic morphology and damage patterns of cavitation damage were revealed by scanning electron microscopy.
• The dynamic evolution characteristics of cavitation bubbles with different structures were elucidated.
• Different modal energy distributions in the jet cavitation field were revealed by dynamic mode decomposition.

## 1 Introduction

Water jet technology offers low wear, structural simplicity, low energy use, and environmental sustainability[1]. These advantages provide great engineering benefits for waterjets, especially in marine engineering applications such as deep-sea mining[2,3], drilling[4], and ship cleaning[5,6]. Self-excited oscillating cavitating jets exhibit self-sustained oscillations through a feedback mechanism of periodic pressure fluctuations. Petal-shaped jets are formed by modifying the nozzle geometry to induce petal-like flow patterns. Engineering reliability and adaptability were improved through interdisciplinary integration.

The cavitation behavior and mechanism of the jet were investigated through visualization experiments and numerical simulations. Wu et al.[7] observed the dynamic evolution of the cavitation cloud in the Helmholtz oscillating cavity using high-speed photographic images and revealed the cavitation mechanism. Zhang et al.[8] investigated the effect of structural parameters of Helmholtz nozzles on impact performance. The frequency-shock coupling mechanism based on the resonance effect theory was revealed by the research results. Shi et al.[9] revealed the relationship between the outlet size of organ pipe nozzles and the pressure-frequency characteristics. Zhang et al.[10] demonstrated that increasing the water jet flow rate and nozzle diameter could induce salt rock crack damage and enhance erosion efficiency. Sun et al.[11] combined experimental fluid displays and computational fluid dynamics to elucidate the generation mechanism and development of cavitation. It was found that high periodicity was exhibited in cavitation morphological changes, which were categorized into three phases: rejoining, leaving, and approaching. Nie et al.[12] employed a heuristic prediction algorithm to predict gas precipitation performance in self-excited oscillating cavities and established parameter intervals for maintaining optimal gas precipitation performance. Yuan et al.[13] performed an in-depth investigation on self-excited oscillating pulsed water jets and developed an innovative locking-frequency prediction model to optimize the performance of self-excited cavitating water jets. Baghaei et al.[14] employed numerical simulations to reveal the dominant influence of nozzle structural parameters on the frequency characteristics of swept cavitation jets. Fang et al.[15,16] observed the cavitation bubble structure in a Helmholtz oscillating cavity using Large Eddy Simulation (LES). The evolution of the cavitation bubbles was divided into four stages: initiation, development, collision and collapse. The results showed that the frequency characteristics of pressure pulsations inside the cavity were influenced by the cavity diameter ratio.

The hydraulic performance of the jet was directly dictated by the nozzle shape[17]. E. Rouly et al.[18] investigated the effect of geometric features in the nozzle internal geometry on jet dispersion angle and fluid distribution using a high-pressure low-dispersion jet nozzle configuration. Li et al.[19] investigated the flow characteristics of different nozzle jets by analyzing the near-field surge structure of the jet. In the analysis of mixing characteristics, it was found that the largest mixing area and the best mixing characteristics were exhibited by the elliptical nozzle jet. Fang et al.[20] compared the jet characteristics of Helmholtz nozzles, Organ pipe nozzles, and Venturi nozzles using large eddy simulation, demonstrating that Helmholtz nozzles exhibit the strongest periodic oscillation effects while Venturi nozzles demonstrate optimal dispersion performance. Yang et al.[21] experimentally tested venturi tubes with different divergence angles and found that the risk of erosion caused by multiscale cavitation behavior increases with decreasing divergence angle. Shakouchi[22] analyzed the flow characteristics of orifice-type, resonance-type, and petal-type nozzles, experimentally demonstrating that petal-type nozzles exhibit optimal mixing, diffusion, and heat transfer properties. Furthermore, he characterized the vortex structure of petal-type jets and systematically elucidated the flow dynamics, mixing behavior, and diffusion mechanisms in petal-shaped nozzles. Rao et al.[23,24] decomposed petal-shaped jet flow images and extracted the jet width growth rate characterizing mixing rate. They found that the petal-shaped nozzle achieves the highest mixing enhancement of 430 % and revealed the three-dimensional flow structure of this nozzle via visualization experiments.

To systematically analyze the coupling mechanism between nozzle structure and cavitation impact performance, a full understanding of the water jet induced crushing mechanism on specimens and its penetration ability is essential. Scanning electron microscope (SEM)[25] and CT scanning[26] are commonly employed to observe microscopic damage patterns and macroscopic rupture patterns in specimens, respectively. Huang et al.[27] measured the dynamic strain of shale specimens after being crushed with high-pressure abrasive water jets. Through SEM analysis, they demonstrated that the intense squeezing pressure of the abrasive jet and edge rupture effects readily generate deep rock fractures. Ji et al.[28] employed self-excited oscillating cavitation water jet (SOCW) technology to crush concrete and elucidated the crushing mechanism of SOCW in submerged environments using SEM and CT, providing critical references for crushing efficiency enhancement. Cao et al.[29,30] investigated the damage morphology and rupture mechanism of rocks under UHP water jet action using CT scanning, and revealed the evolution patterns of their pore structure induced by UHP water jets. Jing et al.[31,32] employed polarized light microscopy to effectively observe mineral damage patterns along with crack sprouting and their developmental processes, providing a powerful complementary approach to SEM and CT for analyzing rock damage mechanisms.

Dynamic Mode Decomposition (DMD) was proposed by Schmid[33]. DMD is a data-driven method for modal analysis of nonstationary flow fields to analyze the main flow features and reconstruct dynamic processes in flow fields[34]. Rowley et al.[35] examined the relationship between the DMD algorithm and Koopman modal decomposition, and successfully generalized this algorithm to nonlinear flows. Ge et al.[36] identified vortex structures with symmetric features in the flow field using the DMD method, and revealed the distinctive flow features causing jet vortex instability. Wang et al.[37] employed the DMD method to identify the top five modes based on energy share ranking for swept cavitation jets. They detected antisymmetric vortex structures in the high-energy modes and characterized the dynamic evolution of the cavitation cloud through low-energy modes. Zhu et al.[38] employed DMD and POD to identify flow patterns in turbulent cavitation flows. Their results indicated that the most energetic structures in the flow field were successfully extracted by POD modes, whereas shedding and collapsing behaviors of cavitation were identified by DMD through frequency-domain decoupling. Liu et al.[39] analyzed the proposed order structure of cavitation flow around a hydrofoil using DMD and POD, demonstrating that DMD decomposes the complex flow field into uncoupled proposed order structures more effectively than POD.

The oscillatory cavitation and diffusion properties of jets could significantly enhance their impact performance. However, due to their fundamental differences in inducing shock damage, distinct damage morphologies and rupture modes emerge. Understanding these differences is crucial for elucidating the structure-cavitation shock coupling mechanism. This study aims to enhance the cavitation fragmentation performance of jets by integrating underwater erosion experiments with large eddy simulations to investigate the influence of petal-shaped nozzle structural parameters on cavitation impact characteristics. A multiscale analysis method integrating cavitation impact and damage mechanisms, along with a metric for cavitation intensity, has been established to quantify the cavitation impact performance of jets. The microscopic fracture morphology of sandstone was characterized using SEM. The cavitation data of the jet was processed by dynamic mode decomposition (DMD). Key dynamics of the cavitation jet were accurately characterized. The mechanism of coupled interaction between nozzle structural parameters and jet cavitation impact was systematically revealed. The research elucidated the interaction mechanisms between nozzle structure and cavitation impact and provided theoretical foundations for optimizing nozzle designs in submerged rock-breaking applications.

## 2 Experimental setup

### 2.1 Sample preparation

Sandstone is the main surface rock of marine resource reservoirs[40]. The experimental samples were obtained from sandstone sourced from Xuzhou City, Jiangsu Province, China, with physical properties detailed in Table 1. Before experiments, the rock core was processed into standard 50 × 50 mm cylindrical specimens, and end faces were polished to achieve the required flatness.

**Table 1:** Sandstone physical properties.

Parameters	Sandstone
Density (g/cm^3^)	2.41
Uniaxial compressive strength (MPa)	27.49
Tensile strength (MPa)	0.73
Elastic modulus (GPa)	8.1
Poisson’s ratio	0.24
Porosity (%)	10.17

### 2.2 Experimental design

As shown in Table 2, erosion experiments were conducted under submerged conditions using two types of cavitation jet nozzles: a petal-shaped nozzle with 27° diffusion angle (hereafter referred to as P.N_27_) and an organ pipe nozzle with 40° diffusion angle (hereafter referred to as O.N_40_). The experimental operating pressure was set to *P* = 16 MPa with erosion duration *T* = 60 s. The variable erosion target spacing *L*/*D_e_* was tested within 2–12 to assess the effects of nozzle structure and target spacing on sandstone cavitation erosion in submerged environments.

**Table 2:** Experimental design.

Nozzle type	Working parameters	Symbol	Unit	Variable settings
P.N_27_	Time	*T*	s	60
	Pressure	*P*	MPa	16
O.N_40_	Standoff distance	*L*/*D_e_*	−	2 4 6 8 10 12

### 2.3 Water jet rock breaking test system

**Fig. 1:**
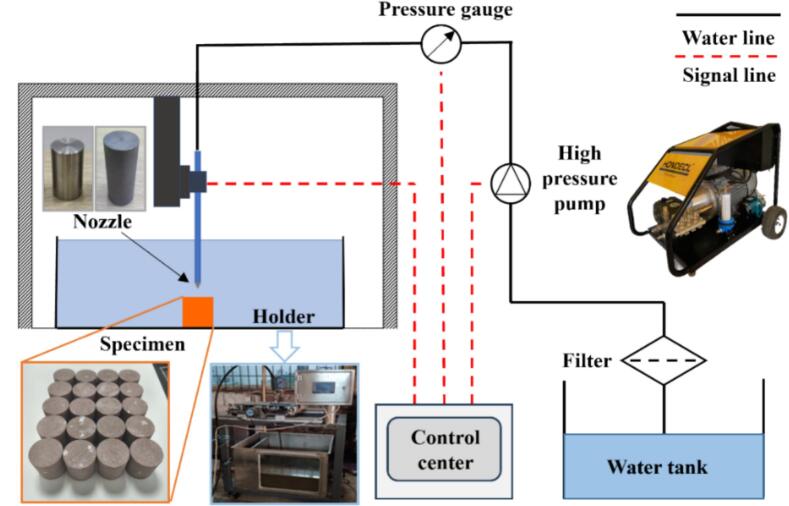
Experimental apparatus of the rock erosion test.

Fig. 1 depicts the experimental testing system for cavitation jet impact under submerged conditions[41]. The main components of the system are connected by means of rational connections in order to realize the cavitation jet impact program. To simulate a submerged environment, the sandstone **is** placed in a tank filled with water. The filtered water is pumped by a high-pressure pump through a high-pressure hose to the inlet of the nozzle. The high-pressure water is converted by the cavity structure of the nozzle into an oscillating cavitation water jet. The water jet flows through the nozzle outlet and into the pool, causing damage to the sandstone. The working pressure of the water jet is controlled by a high-pressure pump and monitored via a pressure gauge. A detachable steel pipe connects the cavitation nozzle to the high-pressure hose. Precise control of the impact target distance is achieved through axial movement.

Erosion depth was measured by a depth sounder and mass loss was determined from mass difference before and after testing. Eroded end face morphology was acquired via image processing for determining the erosion area. Experimental errors mainly resulted from instrument accuracy limitations and procedural inconsistencies. The pressure gauge featured a range of 0–40 MPa and an accuracy class of 1.6; target distance and time measurement accuracies were 0.1 mm and 0.2 s, respectively. To ensure reliability, all measurements were repeated at least three times and averaged.

**Fig. 2:**
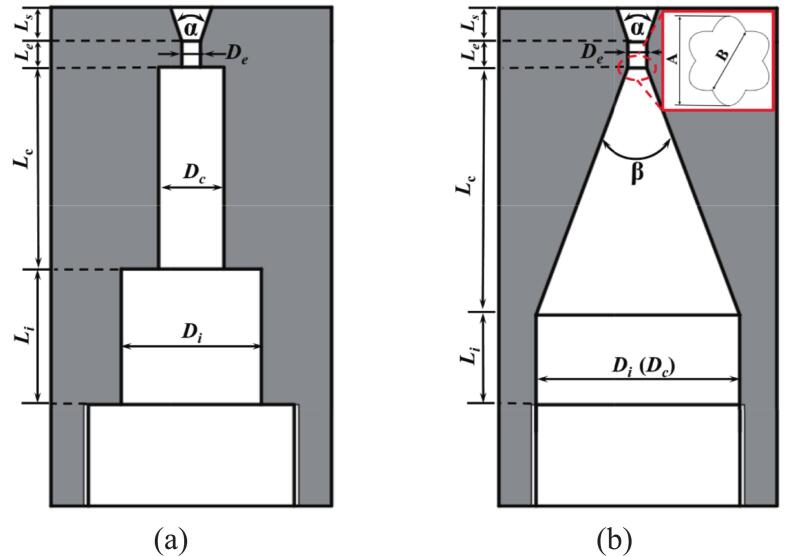
Schematic diagram of nozzle structure. (a) O.N_40_; (b) P.N_27._

The cavitation water jet nozzle was regarded as the most critical component of the entire system, as the outcome of rock breaking across multiple dimensions was significantly governed by its cavitation impact performance and diffusion characteristics. Table 3 presents the detailed parameters of the six nozzles examined in this study. Fig. 2 illustrates the geometry and dimensions of the experimental O.N_40_ and P.N_27_ nozzles. Of these, the petal-shaped nozzle prototype integrates a Venturi tube with an outlet cross-section optimized into a petal-shaped configuration. The petal cross-section design follows previous work[42], featuring a transition angle *β* = 27° as shown in Fig. 2(b). The elliptical cross-section demonstrates alternating axes with the long axis (*A* = 1.1 mm) and short axis (*B* = 0.84 mm) repeating at 30° angular intervals. According to Zhang et al.[43], the throat thickness of the petal-shaped nozzle exit was defined as *L_e_* = 2.0 mm to facilitate the full development of the jet.

**Table 3:** Nozzle dimensions.

Nozzle type	*α*	*D_e_* (*mm*)	*D_i_/D_e_*	*D_c_/D_e_*	*L_i_/D_e_*	*L_c_/D_e_*	*L_e_/D_e_*	*L_s_*/*D_e_*
O.N_27_	27°	1	15	7	10	23	2	3
O.N_40_	40°							
O.N_60_	60°							
P.N_27_	27°	1	15	15	10	33	2	3
P.N_40_	40°							
P.N_60_	60°

### 2.4 Numerical methods and model validation

Cavitation bubble evolution and jet mainstream diffusion are significantly influenced by the diffusion angle of the nozzle. To investigate the influence mechanism of nozzle structure on cavitation characteristics and diffusion behavior, numerical simulations of the cavitation jet impact flow field were conducted using the LES model and the Zwart-Gerber-Belamri (ZGB) model. The model configuration aligns with prior validated work [42], with numerical schemes and solution methodologies detailed in Table 4.

**Table 4:** The numerical simulation scheme.

Grid	Staggered grid
Pressure-velocity coupling algorithm	Coupled
Multiphase model	Mixture
Primary phase	Water-liquid
SGS model	WALE
Viscous terms	Bounded central differencing
Time discretization	Second-order implicit
Time step size, Δt	5 × 10^−6^

**Fig. 3:**
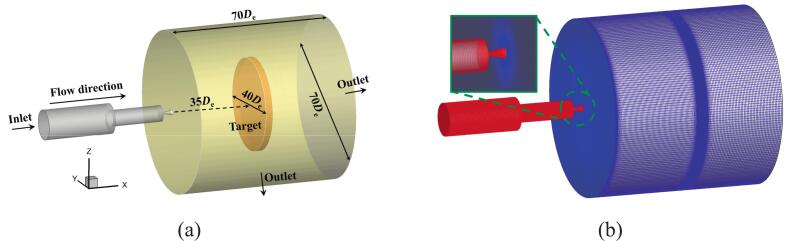
Calculation domain settings. (a) Boundary conditions; (b) Mesh setting.

**Fig. 4:**
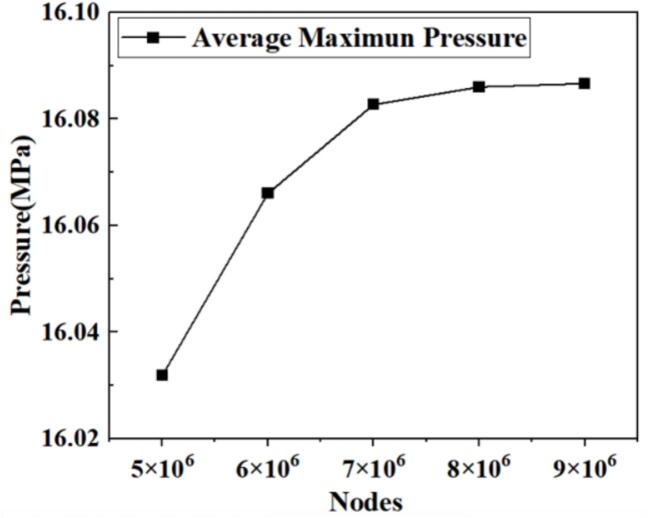
Verification of grid independence.

**Fig. 5:**
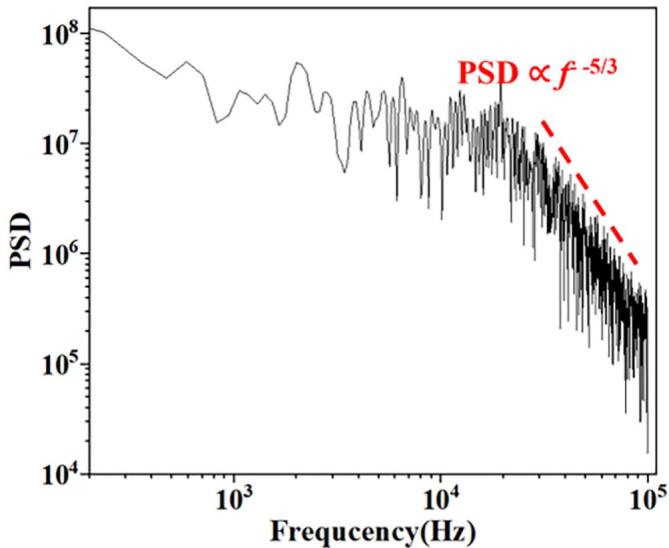
Power spectral density.

The boundary conditions are configured per Fig. 3(a). Grid generation represents a pivotal step in numerical simulations governed by the computational domain. The mesh is divided by a hexahedral mesh as shown in Fig. 3(b). Using O.N_27_ as an example, grid independence was verified using the average peak-to-valley amplitude of 15 consecutive fluctuations at 16 MPa nozzle inlet pressure (Fig. 4). The difference between 8 × 10⁶ and 9 × 10⁶ grids is negligible. The outlet pressure power spectral density (PSD) appears in Fig. 5. The power spectral density within the inertial subrange follows a −5/3 power law, indicating that 8 × 10⁶ grid points are sufficient to accurately predict the −5/3 power law of the pressure spectrum. Consequently, approximately 8 × 10⁶ grids were employed for subsequent studies.

To discuss the erosion strength of cavitation jets at different target spacings, a jet power *P_j_* was proposed to characterize this parameter, defined as [44]:

(1) $$P_{j}=Q\Delta p=Q\left(p_{1}-p_{2}\right)$$

Where *Q* is the flow rate of the jet. *p*_1_ and *p*_2_ are the pressures upstream of the nozzle and at the target point of the flow field, respectively, where the absolute pressure is determined by averaging over 10 consecutive measured peaks.

The erosion experiments employed sandstone mass loss to characterize the cavitation jet erosive strength. As sandstone mass loss is affected by measurement errors and material inhomogeneities, erosion depth and erosion pore area were used to estimate this mass loss and optimize direct measurements. To enable more direct and efficient comparison between experimental and numerical simulation results, the sandstone mass loss and jet power were normalized using average values.

### 2.5 DMD method

After the numerical simulation, a matrix of N flow field snapshot sequences *X*  = [ *x*_1_ *x*_2_ *x*_3_ …*x*_N_] is obtained, where the time interval *Δt* between adjacent snapshots *x_i_* and *x_i_*_+1_ is small. The vectors *x_i_* and *x_i_*_+1_ are linearly mapped:

(2) $$x_{i+1}=Ax_{i}$$

Where *A* is a high-dimensional matrix.

The number of snapshots is much smaller than the number of cross-section nodes. The eigenvalues are estimated in descending order of the matrix *A*. Submatrices $X_{1}^{N-1}=\left[x_{1}\;x_{2}\;x_{3}\;...{\text{x}}_{\text{N - 1}}\right]$ and $X_{2}^{N}=\left[x_{2}\;x_{3}\;x_{4}\;...{\text{x}}_{\text{N}}\right]$ are constructed and substituted into Eq. (2) according to the following relationship:

(3) $$X_{2}^{N}=\left[x_{2}\;x_{3}\;x_{4}...x_{N}\right]=\left[Ax_{1}\;Ax_{2}\;...Ax_{N-1}\right]=AX_{1}^{N-1}$$

(4) $$X_{1}^{N-1}=U\Sigma V^{*}$$

(5) $$A\approx U\tilde{A}U^{*}$$

Where *U* is the singular matrix and U* is its complex conjugate transpose. Σ is the singular-valued diagonal matrix and V* is the complex conjugate transpose matrix of *V*.

The singular values of the matrix $X_{1}^{N-1}$ are decomposed by Eq. (4). The best low-dimensional approximation matrix $\tilde{A}$ of *A* was constructed using the similarity principle.

(6) $$\tilde{A}=U^{*}X_{2}^{N}V\Sigma^{-1}$$

The diagonal matrix *Λ* consisting of *λ_i_* and the eigenvector matrix consisting of *ξ_i_* can be computed by Eq. (6). The logarithm of the eigenvalue *λ_i_* is given by the following equation:

(7) $$\frac{\ln\lambda_{i}}{\Delta t}=\sigma_{i}+j\omega_{i}=\frac{\ln\left|\lambda_{i}\right|}{\Delta t}+j\frac{\arg\lambda_{i}}{\Delta t}$$

Where *σ_i_* is the growth rate of the *i_th_* mode and *ω_i_* is the frequency information of the *i_th_* mode.

The frequency is

(8) $$f_{i}=\frac{\omega_{i}}{2\pi }$$

## 3 Results and discussion

### 3.1 Macro-erosion morphology analysis

**Fig. 6:**
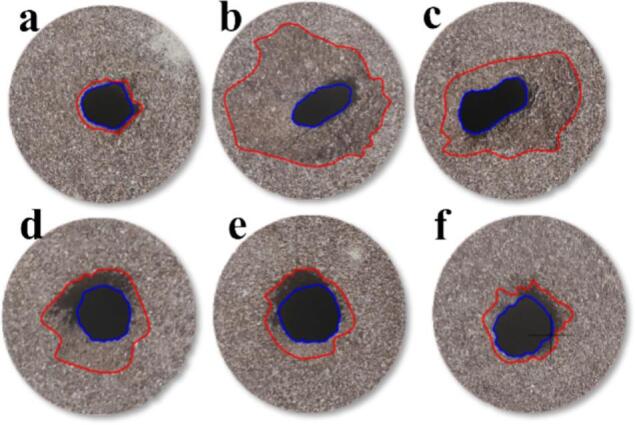
Macroscopic morphology of sandstone under O.N_40_ cavitation impact. *P* = 16 MPa; *T* = 60 s; (a)*L*/*D_e_* = 2; (b)*L*/*D_e_* = 4; (c)*L*/*D_e_* = 6; (d)*L*/*D_e_* = 8; (e)*L*/*D_e_* = 10; (f)*L*/*D_e_* = 12.

O.N_40_ generates a typical self-excited oscillating cavitation jet with field modulation through structural parameter adjustments. P.N_27_ incorporates a Venturi prototype with an outlet cross-section optimized to petal-shaped geometry, producing a cavitation jet with enhanced diffusion properties. Fig. 6 displays the macroscopic morphology of sandstone under O.N_40_ exposure. Erosion pit geometry becomes progressively rounded with increasing target distance. Notably, extensive damage zones emerge on sandstone surfaces at *L*/*D_e_* = 4 and 6. The self-oscillating cavitation jet, functioning as a hybrid jet type, exhibits elevated instantaneous peak impact forces that enhance erosion generation. Concurrently, when water hammer pressure exceeds the rock uniaxial compressive strength, it induces shallow surface fragmentation through transient water hammer effects[45]. These dual mechanisms collectively produce extensive sandstone surface damage.

**Fig. 7:**
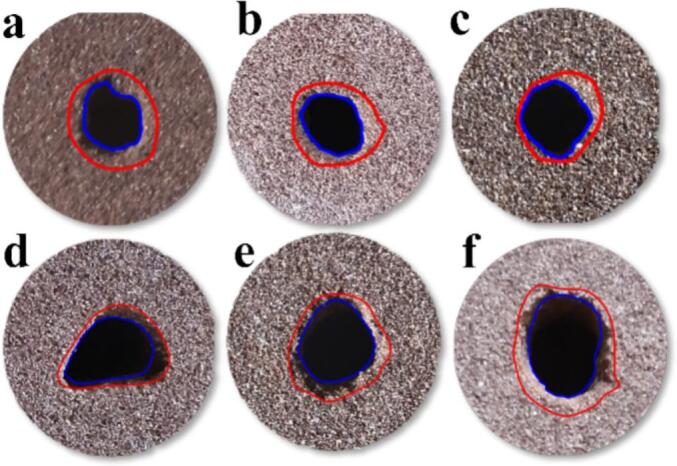
Macroscopic morphology of sandstone under P.N_27_ cavitation impact. *P* = 16 MPa; *T* = 60 s; (a)*L*/*D_e_* = 2; (b)*L*/*D_e_* = 4; (c)*L*/*D_e_* = 6; (d)*L*/*D_e_* = 8; (e)*L*/*D_e_* = 10; (f)*L*/*D_e_* = 12.

Fig. 7 presents the macroscopic morphology of sandstone eroded by P.N_27_. The erosion crater morphology transitions to irregular shapes with increasing target distance. This is attributable to the extended wetted perimeter of the petal-shaped nozzle that promotes large-scale cavitation bubble generation and enhanced jet diffusion. Edge flow field instabilities are amplified by this nozzle geometry, resulting in non-uniform circumferential jet expansion.

**Fig. 8:**
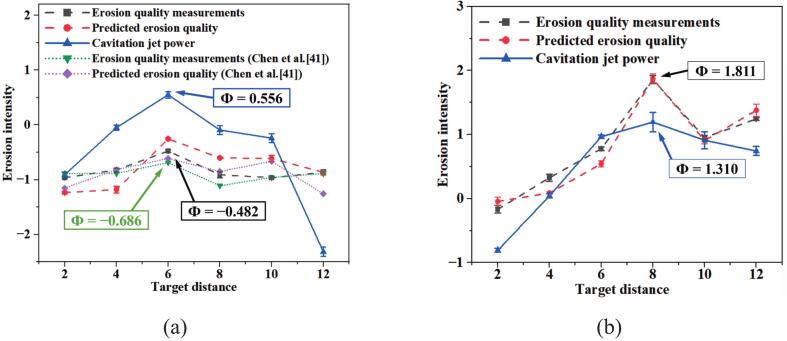
Erosion intensity and cavitation jet power at different target distances. (a)O.N_40_; (b)P.N_27_.

Fig. 8 shows the variation of cavitation erosion strength for O.N_40_ and P.N_27_ at different target spacings. The experimental erosion strength is represented by the mass loss from direct measurements. The cavitation jet power is derived from numerical simulations. Targets are positioned on the flow field central axis as illustrated in Fig. 3(a). Fig. 8(a) references the findings of Chen et al.[41], ensuring consistency in nozzle structure and jet conditions, and normalizes the data. As the target distance increases, the cavitation erosion intensity exhibits an initial increase followed by a decrease. The trend of erosion intensity and the optimal target spacing characterized by the cavitation jet power are in perfect agreement with the experimental results. It is shown that the LES model and the ZGB cavitation model can accurately predict cavitation erosion intensity.

Specifically, O.N_40_ achieves its maximum erosion intensity at a target spacing of *L*/*D_e_* = 6, consistent with the findings of Chen et al.[41]. P.N_27_ achieves peak erosion intensity at a target distance of *L*/*D_e_* = 8. This is because when the target distance is short, damage results from direct fracturing by the main jet, while bubbles are fewer and underdeveloped, struggling to inflict significant damage on the sandstone. At a target distance of *L*/*D_e_* = 2, the sandstone erosion pore area is minimized under jet action and is comparable to the damaged area. When the target distance increases, bubble quantity rises and they fully develop, enabling the jet to strike with peak force and its greatest destructive capability. At the optimal target distance, the sandstone erosion hole area increases by 35 % under the action of O.N_40_, with erosion intensity rising by 48 %. Under the action of P.N_27_, the sandstone erosion hole area increases by 42 %, and erosion intensity surges by 198 %. However, P.N_27_ tends to induce larger-scale bubbles, requiring greater target spacing for full bubble development. This results in a larger optimal target spacing for P.N_27_ compared to O.N_40_, along with higher erosion intensity. Specifically, the maximum erosion mass loss is 1.64 times that of O.N_40_, and the maximum erosion intensity is 2.29 times greater than that of O.N_40_. Thereafter, further increasing the target spacing results in rapid dissipation of the jet mainstream energy due to backflow effects and pits. Rock debris is driven by the jet to continuously erode the pit wall, while significant energy loss occurs along the water jet axis, and rock-breaking capacity is gradually diminished.

It is noteworthy that at a target distance of *L*/*D_e_* = 12, the cavitation jet power of O.N_40_ shows a significant decrease, but its erosion mass decreases only slightly. This is caused by the particle rebound effect. After the jet strips small particles from the sandstone, the greater target distance provides favorable conditions for flow field mixing. The jet drives the particles to collide with the erosion pit wall, partially compensating for the main flow energy loss. In contrast, P.N_27_ exhibits a more uniform erosion pattern, with erosion intensity showing a rebound phenomenon. This indicates that P.N_27_ possesses superior cavitation bubble generation capability compared to O.N_40_, explaining the slower jet power decay for this nozzle structure. With the excellent diffusion and mixing capabilities of the petal jet, the rebound effect of the particles is significantly enhanced and causes secondary damage to the eroded pore structure.

### 3.2 Sem-based identification of cavitation impact damage in sandstones

**Fig. 9:**
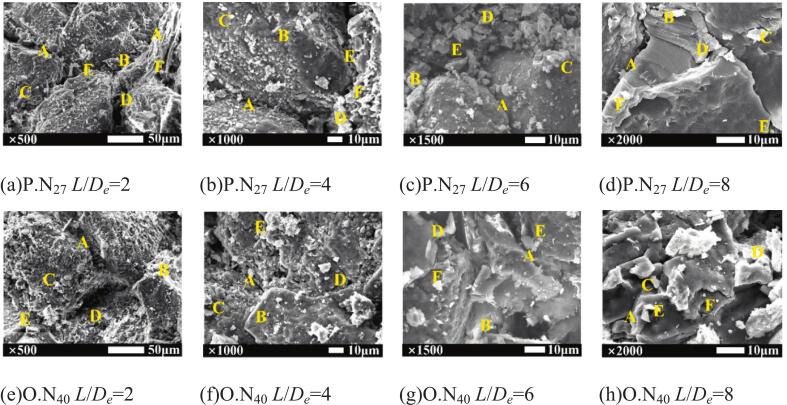
Scanning electron micrographs of eroded surfaces. *P* = 16 MPa, *T* = 60 s. In these figures, “A” denotes microcracks; “B” denotes intergranular fractures; “C” denotes transgranular fractures; “D” denotes pores; “E” denotes wedge-shaped inserts; “F” denotes lamellar inserts.

To investigate the microscopic features and damage patterns after cavitation impact, scanning electron microscopy (SEM) was used to observe the surface morphology of the sandstone [46,47]. Fig. 9 shows microstructures in pit sidewalls and damage areas following cavitation impact by P.N_27_ and O.N_40_ jets at target distances *L*/*D_e_* = 2, 4, 6, and 8, with magnifications of 500×, 1000×, 1500×, and 2000 × respectively.

At *L*/*D_e_* = 2, sandstone surfaces eroded by P.N_27_ exhibit higher densities of erosion pits and microcracks (Fig. 9a), manifesting macroscopically as pronounced mottling. This occurs because small target spacing enables direct fracturing by the jet core region. The greater wetted perimeter of the petal-shaped nozzle, combined with its petal-shaped flow geometry, generates multi-scale heterogeneous damage on rock surfaces. In contrast, the O.N_40_ jet delivers homogeneously concentrated energy, resulting in fewer and spatially clustered microcracks. Fig. 9, Fig. 9 show the damage pattern of cavitation bubble erosion, i.e., the appearance of regions with fine pores. This special microstructure forms when sandstone undergoes bubble collapse in a submerged environment. Bubbles collapse upon impact with the sandstone surface and generate a strong pressure differential. Driven by the pressure difference, the ends of the microcracks undergo stretching and the connections between particles are thereby severed. The sandstone is subjected to the combined impact of bubbles and the main jet, further intensifying the damage. The damage manifests as fine-pore regions on the sandstone surface.

It can be seen that the cracks produced by the impact of the P.N_27_ jet are larger and longer than those of O.N_40_, despite the different target spacings. Under the influence of this jet, the water in the pore space is displaced by the strong mixed flow field, while new water enters the pore space. The turnover of pore water has complex effects on sandstone damage. On the one hand, the mineral particles are softened and the particle strength is reduced; on the other hand, the adhesion between particles is weakened. Under the influence of these two factors, numerous cracks form on the sandstone surface. Stress concentration tends to occur at particle-microcrack interfaces, generating a water wedge effect that promotes microcrack propagation [48]. Compared to the mottled surface of sandstone subjected to P.N_27_ dispersed vacuoles and jetting, the surface minerals of sandstone under O.N_40_ exhibit tighter bonding with the matrix and sustain less damage from minimal rock debris.

A mixed damage pattern comprising intergranular and transcrystalline fractures was repeatedly observed in rock samples. The optimal standoff distances are *L*/*D_e_* = 8 for P.N_27_ and *L/D_e_* = 6 for O.N_40_, where rock breakage is maximized. In this state, microcracks are squeezed and expanded by the high-pressure water flow and eventually penetrated. Sandstone microcrack walls are crushed by penetrating high-pressure water, and particles between cracks are transported by water molecules. The penetrated microcracks are identified as large smooth sections. The chip remaining in the upper right corner of Fig. 9(d) is the stripping product.

### 3.3 Cavitation flow characteristics

**Fig. 10:**
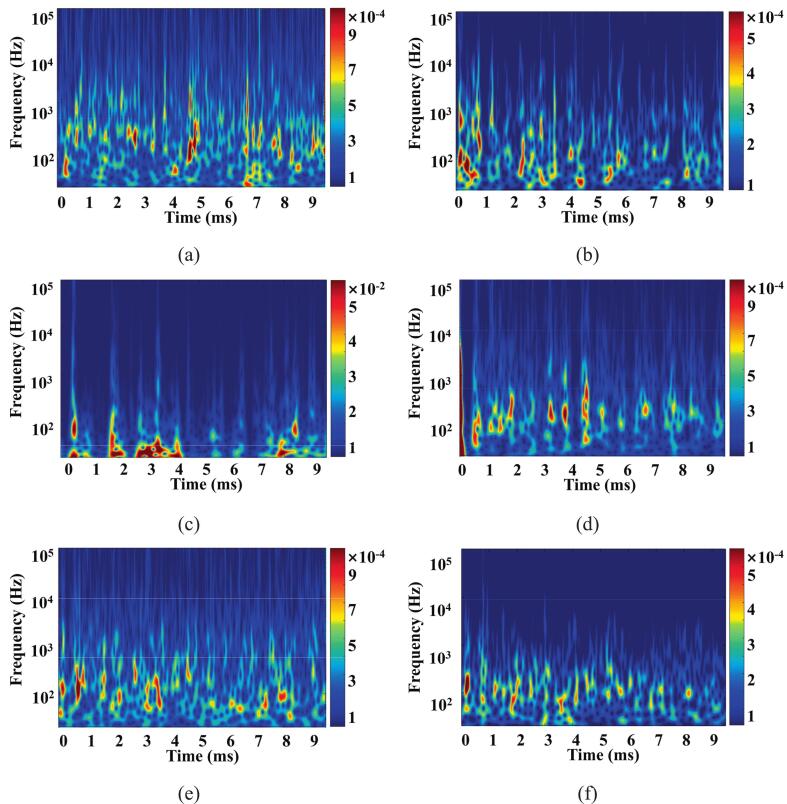
Time-frequency plots of pressure at the outlet for different configurations. (a)O.N_27_; (b)P.N_27_; (c)O.N_40_; (d)P.N_40_; (e)O.N_60_; (f)P.N_60_;

Cavitation is the phenomenon in which small bubbles or nuclei in a liquid are rendered unstable and rapidly expand due to a sudden change in pressure [49]. Energy pulsations in the cavitation cloud structure are responsible for inducing significant pressure pulsations at the nozzle outlet. The time-varying characteristics of the instantaneous frequency of the pressure signal were further analyzed using the Morlet wavelet transform[50]. Fig. 10 shows the time–frequency plot of the pressure at the outlet for different nozzle configurations. From the figure, it can be observed that the non-stationary flow of fluid at the outlet exhibits a pronounced time dependence, and nozzles with different structural parameters demonstrate significant differences in transient frequency. For the 27° diffusion angle structure, the transient pressure pulsations of P.N_27_ are primarily concentrated within the 50–1000 Hz range. In contrast, the cavitation jet of O.N_27_ exhibits a significant energy distribution in the mid-frequency band centered around 200 Hz.

O.N_40_ exhibits a narrower pressure signal bandwidth when the diffusion angle is increased from 27° to 40°. However, P.N_40_ exhibits the opposite trend. It is noteworthy that their energy amplitudes in the middle and low frequency bands are increased, with the O.N_40_ increase being the most significant by two orders of magnitude, and the energy is concentrated below 100 Hz. Since the energy amplitude of the pressure pulsation is positively correlated with the cavitation intensity, when the cavitation intensity is higher at the nozzle outlet, the outlet pressure pulsation shows a low-frequency pulsation with a higher energy amplitude. This indicates that the structure of O.N_40_ is more conducive to maintaining energy stability and possesses better cavitation shock generation capability. When the diffusion angle increases to 60°, the high-energy instantaneous pressure pulsation of O.N_60_ expands sharply into the high-frequency band and loses the ability to induce air bubbles. Fig. 10(f) shows that the cavitation performance of the petal-shaped nozzle structure enhances significantly as the diffusion angle continues to increase, resulting in the P.N_60_-generated jet exhibiting excellent cavitation impact performance.

**Fig. 11:**
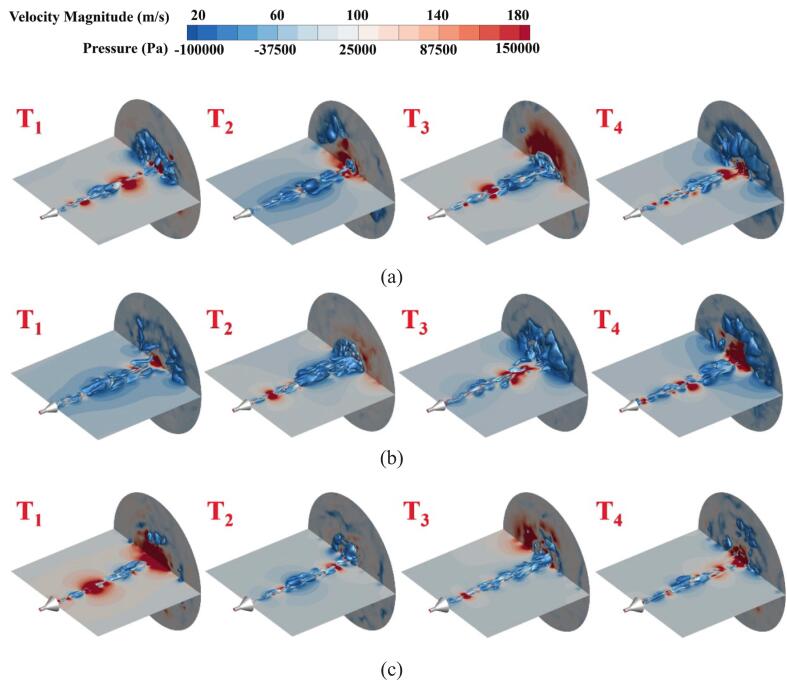
Evolution of cavitation clouds in organ-pipe structures and pressure distribution in shock flow fields. The vapor volume fraction is 25 % equivalent surface. The time interval is 0.2 ms. (a)O.N_27_; (b)O.N_40_; (c)O.N_60_.

Fig. 11 shows the evolution of the cavitation cloud, expressed as 25 % gas volume fraction equivalent surfaces, and the pressure distribution in the flow field for jets of O.N_27_, O.N_40_, and O.N_60_. Under the influence of pressure drop, numerous small-scale bubbles at the nozzle outlet begin to expand and propagate downstream at the T_1_ moment. At the T_2_ moment, the periodicity of nozzle-generated cavitation bubbles becomes evident. Additionally, cavitation bubble shape changes due to vorticity effects and viscous stretching, accompanied by outwardly propagating pressure waves in the flow field. By the T_3_ moment, the cavitation bubble is significantly stretched and expanded. During this process, the bubble collapses upon impacting the target surface, causing the pressure at the impact point to rise significantly. At the T_4_ moment, cavitation clouds sequentially impinge on the target surface while the structure undergoes outward expansion across the target surface. This expansion induces significant energy dissipation that triggers cavitation cloud collapse, creating localized high-pressure zones on the target surface.

The incipient cavitation clouds of O.N_27_ and O.N_60_ are primarily characterized by small-scale bubbles, rendering them more susceptible to energy dissipation-induced collapse during development. This leads to rapidly rising local pressures within the flow field. Among these, O.N_60_ exhibits the most pronounced cavitation bubble collapse behavior. The O.N_40_ initial cavitation bubbles exhibit a more concentrated structure and demonstrate stable stretching and expansion during downstream development. Although minor bubble collapse occurs, it has no significant impact on main bubble development, indicating the 40° diffusion angle better maintains cavitation bubble energy and ensures superior shock pressure generation capability.

**Fig. 12:**
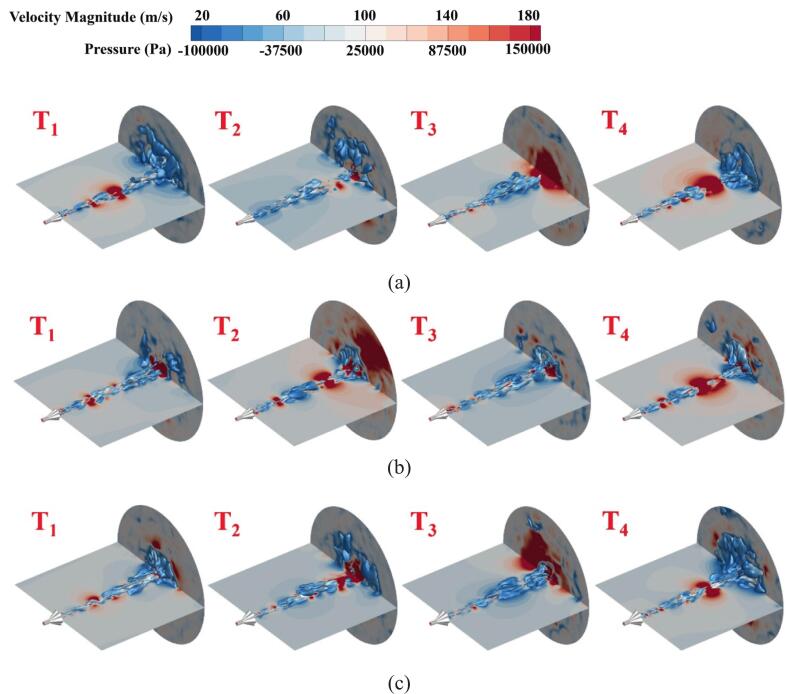
Evolution of cavitation clouds with petal-shaped structures and pressure distribution in shock flow fields. The vapor volume fraction is 25 % equivalent surface. The time interval is 0.2 ms. (a)P.N_27_; (b)P.N_40_; (c)P.N_60_.

Fig. 12 shows the evolution of the cavitation cloud, expressed as 25 % gas volume fraction equivalent surfaces, and the pressure distribution in the flow field for jets of P.N_27_, P.N_40_, and P.N_60_. Unlike the organ-pipe structure, the petal-shaped nozzle generates incipient cavitation bubbles with larger-scale structures and improved dispersion characteristics. The cavitation bubbles of P.N_27_ and P.N_40_ frequently experience large-bubble collapse during downstream development, manifesting as multiple pressure surge regions in the flow field—consistent with phenomena observed in pressure pulsation spectrograms. P.N_60_ cavitation bubbles exhibit significantly accelerated development speed and expanded range, with higher density, generating stronger localized pressures upon collapse at the target surface.

### 3.4 DMD analysis

**Fig. 13:**
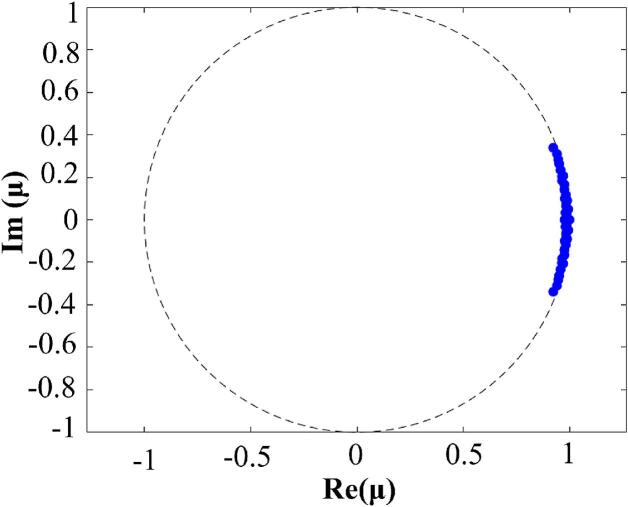
Eigenvalue distribution.

After the cavitation jet had stabilized, the vapor volume fraction data from the X-Y sections of the six structural configurations were analyzed using the DMD method. The resulting modal eigenvalue distributions are presented in Fig. 13. The quasi-periodic oscillatory characteristics of the cavitating jet manifest through conjugate pairs of modal eigenvalues distributed along the unit circle. The quasi-periodic dynamics of cavitation phenomena in organ-pipe and petal-shaped nozzle configurations are confirmed.

**Fig. 14:**
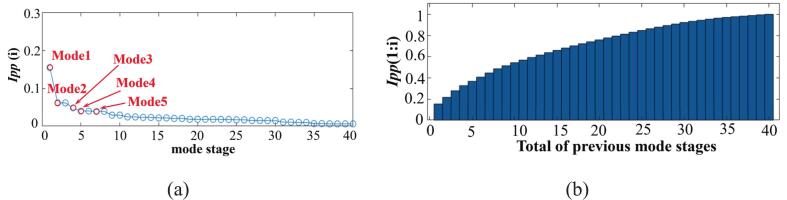
Modal energy share distribution. (a) Energy share of the *i_th_* mode. (b) The total energy of *i* modes.

After flow field decomposition, selecting dominant modes is a key step for reduced-order reconstruction and prediction. Each mode makes a distinct energy contribution to the cavitation flow field. Fig. 14 shows the modal energy percentage and the cumulative modal energy, sorted from largest to smallest according to modal energy. Paired eigenmode patterns appear as conjugate modes, representing the same dynamic behavior. Only one of the conjugate modes is used to study the dynamic characteristics. The frequency for each mode, along with the real and imaginary parts of the eigenvalue *λ_i_*, is calculated using Eq. (7), (8). The cumulative energy percentage for *i* modes and the energy share of the *i_th_* mode are calculated using the following equation:

(9) $$\mathrm{Ipp}\left(i\right)=I_{i}/\sum_{i=1}^{R}I_{i}$$

(10) $$\mathrm{Ipp}\left(1:i\right)=\left(\sum_{i=z}^{N}I_{i}/\sum_{i=z}^{R}I_{i}\right)\times 100\%,\;N=1,2,...,R$$

where *R* represents the number of the remaining modes.

The characteristic parameters of the first five modal orders are presented in Table 5. The first-order mode exhibits zero imaginary part and frequency values, while demonstrating the highest energy share at 15.54 %. This eigenmode corresponds to the steady-state cavitation flow field, which remains temporally invariant. The steady-state cavitation flow field demonstrates dominance in bubble dynamics.

**Table 5:** Top five dominant dynamic modal parameters.

Mode	Re*λ_i_*	Im*λ_i_*	Growth Rate	*f_i_/*Hz	Energy Proportion(%)
1	0.9982	0	−168	0	15.54
2	0.9807	0.0328	−3784	6689	6.20
3	0.9868	0.0284	−4749	5753	4.85
4	0.9885	0.0907	−2574	18,299	4.00
5	0.9637	0.2029	−1470	41,508	3.90

**Fig. 15:**
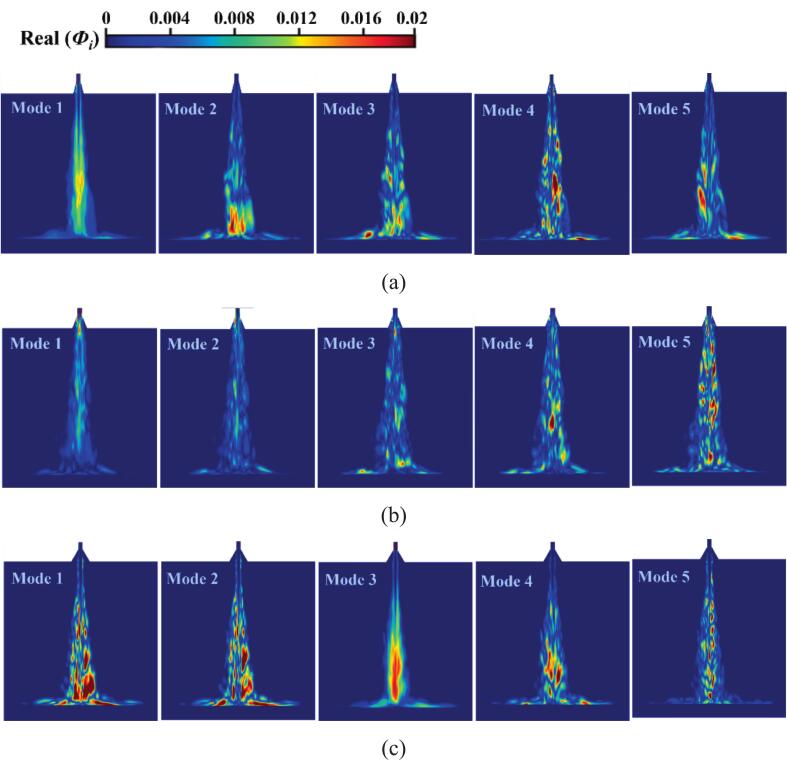
The vapor volume fraction data of the organ-pipe structure was processed by DMD to obtain the first 5 orders of modal plots of the energy distribution ratio. (a)O.N_27_; (b)O.N_40_; (c)O.N_60_.

**Fig. 16:**
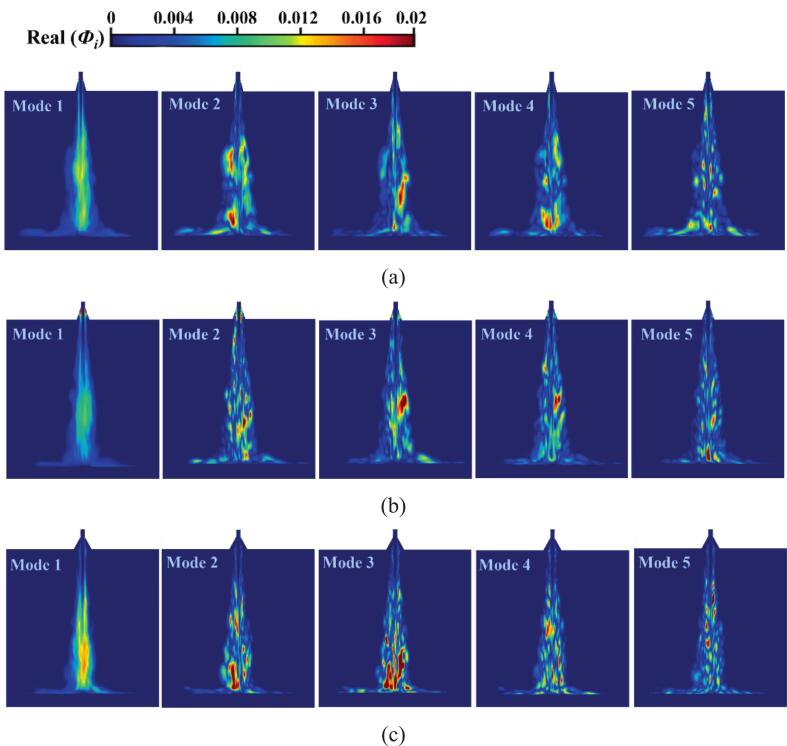
The vapor volume fraction data of the petal-shaped structure was processed by DMD to obtain the first 5 orders of modal plots of the energy distribution ratio. (a)P.N_27_; (b)P.N_40_; (c)P.N_60_.

Fig. 15, Fig. 16 respectively display the first five dominant modal energy distribution plots obtained from DMD-processed cavitation flow field data for the six nozzles. The first mode is identified as the steady-state mode, exhibiting the highest proportion in the energy contribution spectrum. This mode characterizes the essential features of the cavitation flow field, with the mean flow field maintaining temporal invariance. The second- and third-order mode energy contributions primarily originate from bubbles proximal to the nozzle diffusion angle, exhibiting significant proportions in the energy spectrum. Downstream shedding of large-scale bubbles is also revealed by these modes, which are identified as principal coherent structures driving the quasi-periodic cavitation phenomenon of the nozzle. The cavitation bubble structure size diminishes progressively with increasing modal order. Higher-order modal energies constitute smaller proportions of the total energy, while the shedding behavior of numerous small-scale bubbles represents a distinct low-energy exchange event. Although these higher-order modes cannot dominate the time-averaged cavitation flow field, the interaction between frequent cavitation bubble shedding and vortex dynamics governs bubble development within the cavitation jet. These interaction mechanisms establish the connection between dynamic mode decomposition and transient cavitation dynamics.

For O.N_27_ and O.N_60_ cavitation flow fields, initial bubbles primarily develop along the shear layer. The dynamic evolution of cavitation bubbles—including incipient nucleation, stretching, expansion, and collapse phases—and their spatial distribution are captured by second-order modes. O.N_60_ is the only structure where the steady mode appears in the third-order position. Its high-energy modes are governed by numerous small-scale bubbles, and this modal signature of bubble collapse quantitatively characterizes the transient dynamics of the implosion process. Numerous high-energy incipient bubbles are observed along the inner diffusion wall of O.N_40_, maintaining energy stability during development. As the cavitation cloud progresses downstream, uniform expansion velocities are exhibited, and the influence of the diffusion angle on bubble dynamics is demonstrated.

For the cavitation shock flow fields of P.N_27_, P.N_40_, and P.N_60_ obtained via dynamic mode decomposition (DMD), the time-averaged fields are characterized by first-order modes. Specifically, the high-energy regions of P.N_40_ concentrate near the diffuser angle, whereas those of P.N_60_ are primarily governed by large-scale cavitation bubbles downstream. A clearly delineated cavitation bubble evolution process is revealed by the second-order modes. In P.N_27_ and P.N_40_ cavitating flow fields, primary coherent structures experience repeated collapse events within the mid-flow region. In contrast, P.N_60_ dominant coherent structure exhibits extensive collapse after target surface impact, with this spatial localization matching the dominant pressure pulsation frequency characteristics. Cavitation bubbles in higher-order modes manifest as multiple small-scale vortex structures. Although these structures contribute minimally to the total energy spectrum, they directly reveal the spatio-temporal evolution of microbubbles within the shear layer. Marked attenuation in coherent structure strength is observed relative to the first two dominant modes, and small-scale vortex dynamics are primarily governed by these higher-order modes.

## 4 Conclusion

This study systematically investigates the interaction mechanism of nozzle structures and cavitation impact through submerged erosion experiments and large-eddy simulation. The optimal nozzle structural and operating parameters for generating maximized cavitation impacts were determined, providing a theoretical basis for the application of cavitation jet nozzles in submerged rock fragmentation. The following were the principal findings:

(1) In the submerged environment, cavitation jet impact intensity shows an initial increase followed by a decrease with increasing standoff distance. Optimal standoff distances occur at *L*/*D_e_* = 6 for O.N_40_ and *L*/*D_e_* = 8 for P.N_27_, where maximum cavitation erosion intensity and peak rock-breaking effectiveness are achieved. Under the optimal target distance conditions, the sandstone erosion hole areas increased by 35 %, and the erosion intensity rose by 48 % under the action of O.N_40_. Meanwhile, the sandstone erosion hole areas increased by 42 %, and the erosion intensity surged by 198 % under the action of P.N_27_.

(2) The microscopic characteristics and damage patterns of sandstone subjected to P.N_27_ and O.N_40_ cavitation jets were characterized through SEM analysis. The number of erosion pits and microcracks on the surface of sandstone subjected to P.N_27_ cavitation impact was significantly larger. Macroscopically, the surface appeared relatively more mottled. The fine pore zones were a special microstructural region caused by the collapse of submerged bubbles. The sandstone was subjected to the combined impact of bubbles and the main jet, further intensifying the damage.

(3) The evolution of the vortex structures inside the chamber was strongly influenced by the nozzle structure. P.N_60_ demonstrated a more concentrated pressure pulsation energy amplitude, indicating superior shock pressure generation capability. O.N_40_ more effectively maintained energy stability. The cavitation cloud evolution and flow field pressure variations were characterized. Localized high-pressure phenomena in the flow field or on the target surface emerged during cavitation cloud collapse, accompanied by outwardly propagating pressure waves. P.N_60_ cavitation bubbles demonstrated a greater development rate and range with higher density, forming enhanced localized pressures upon collapse at the target surface, generating more substantial impact effects.

(4) The energy modes of the cavitation flow field were identified through DMD analysis. The first mode exhibits the dominant energy contribution and represents the time-averaged feature of the cavitation field. The second-order modes systematically reveal the incipient nucleation, stretching, expansion, and collapse phases of cavitation bubbles along with their spatial distribution. Higher-order modes successfully capture the spatio-temporal evolution of microbubbles within the shear layer.

This study also revealed the limitations of the “erosion flow field” strategy. For CFD-based simulations of jet erosion, obtaining corresponding data measurements for backflow and entrainment erosion within the erosion cavity is challenging. As a result, the “erosion flow field” strategy is better suited for simulating the external flow field and the dynamic behavior of bubbles on the target surface. Since this study conducted sandstone erosion tests in a submerged environment, comparative validation allows for a qualitative assessment of backflow and vortical suction characteristics within the erosion cavity. This may provide valuable insights for future nozzle design research, although further visualization verification is needed. Furthermore, this study only considers the effects of individual factors, which exhibit strong interactions. Future research should focus on multi-objective optimization.

## CRediT authorship contribution statement

**Xiuneng Li:** Writing – review & editing, Writing – original draft, Conceptualization. **Xide Cheng:** Supervision, Conceptualization. **Wenjiang Hou:** Methodology, Investigation. **Shidong Fan:** Conceptualization. **Xiaofeng Guo:** Methodology, Conceptualization. **Xiangshu Lei:** Investigation. **Zhenlong Fang:** Writing – review & editing, Supervision, Methodology, Funding acquisition. **Yan Chen:** Writing – review & editing, Methodology.

## Declaration of competing interest

The authors declare that they have no known competing financial interests or personal relationships that could have appeared to influence the work reported in this paper.
